# Transforming Growth Factor-β: A Multifunctional Regulator of Cancer Immunity

**DOI:** 10.3390/cancers12113099

**Published:** 2020-10-23

**Authors:** Vivian Weiwen Xue, Jeff Yat-Fai Chung, Cristina Alexandra García Córdoba, Alvin Ho-Kwan Cheung, Wei Kang, Eric W.-F. Lam, Kam-Tong Leung, Ka-Fai To, Hui-Yao Lan, Patrick Ming-Kuen Tang

**Affiliations:** 1 Department of Anatomical and Cellular Pathology, State Key Laboratory of Translational Oncology, The Chinese University of Hong Kong, Hong Kong 999077, China; weiwenxue@cuhk.edu.hk (V.W.X.); jeffchung@link.cuhk.edu.hk (J.Y.-F.C.); cristinagarcia@link.cuhk.edu.hk (C.A.G.C.); acheung@cuhk.edu.hk (A.H.-K.C.); weikang@cuhk.edu.hk (W.K.); kfto@cuhk.edu.hk (K.-F.T.); 2 Department of Surgery and Cancer, Imperial College London, Hammersmith Hospital Campus, London W12 0NN, UK; eric.lam@imperial.ac.uk; 3 Department of Paediatrics, The Chinese University of Hong Kong, Shatin, Hong Kong 999077, China; ktleung@cuhk.edu.hk; 4 Department of Medicine and Therapeutics, Li Ka Shing Institute of Health Sciences, The Chinese University of Hong Kong, Hong Kong 999077, China; hylan@cuhk.edu.hk

**Keywords:** TGF-β, cancer immunity, tumour microenvironment

## Abstract

**Simple Summary:**

Transforming growth factor beta (TGF-β) is a multifunctional cytokine that can restrict cancer onset but also promote cancer progression at late stages of cancer. The ability of TGF-β in producing diverse and sometimes opposing effects relies on its potential to control different cellular signalling and gene expression in distinct cell types, and environmental settings. The tumour promoting role of TGF-β is primarily mediated through its effects on the local tumour microenvironment (TME) of the cancer cells. In this review, we discuss the most recent research on the role and regulation of TGF-β, with a specific focus on its functions on promoting cancer progression through targeting different immune cells in the TME as well as its therapeutic perspectives.

**Abstract:**

Transforming growth factor-β (TGF-β) was originally identified as an anti-tumour cytokine. However, there is increasing evidence that it has important roles in the tumour microenvironment (TME) in facilitating cancer progression. TGF-β actively shapes the TME via modulating the host immunity. These actions are highly cell-type specific and complicated, involving both canonical and non-canonical pathways. In this review, we systemically update how TGF-β signalling acts as a checkpoint regulator for cancer immunomodulation. A better appreciation of the underlying pathogenic mechanisms at the molecular level can lead to the discovery of novel and more effective therapeutic strategies for cancer.

## 1. Introduction

Transforming growth factor-beta (TGF-β) is a secretory cytokine that has pleiotropic roles in cancer progression through controlling cell proliferation, differentiation, apoptosis, and migration. The TGF-β family of cytokines consists of three different isoforms, TGF-β1, TGF-β2, and TGF-β3, with each of them having a unique expression mode and executing distinct functions. For example, TGF-β2 can deplete interleukin 6 (IL-6) function and induce apoptosis [1], TGF-β3 affects the differentiation of mesenchymal stromal cells (MSCs) [2], while TGF-β1 plays an important role in cancer progression and tumour microenvironment (TME) development [3,4].

**Figure 1:**
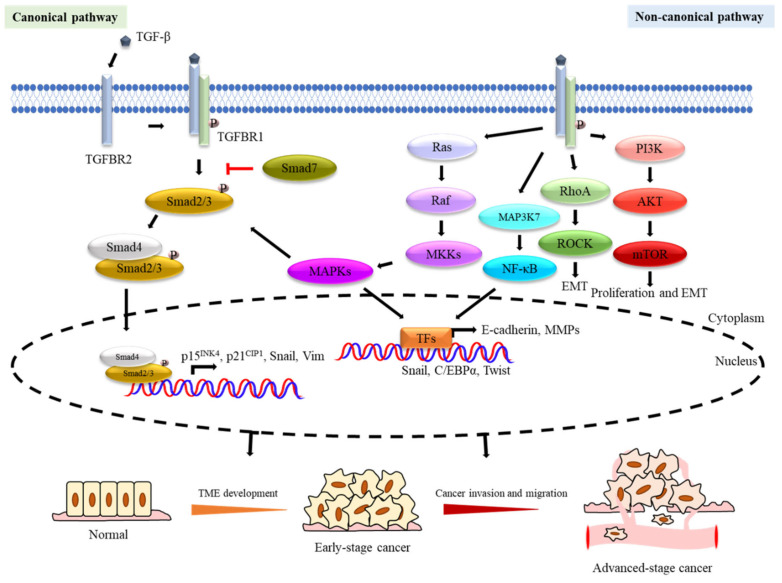
Transforming growth factor-β (TGF-β) signalling pathways in tumorigenesis. The dual roles of TGF-β signalling pathways have been demonstrated in tumorigenesis. TGF-β is a tumour suppressor in TME development of early-stage cancer and a tumour promoter in malignancy processes of advanced-stage cancer. Schematic diagramme (above) showing TGF-β signalling and its role in cancer tumorigenesis and progression as well as tumour suppression. TGF-β binds to TGFBR2 which then complexes with TGFBR1 to activate downstream signalling. TGF-β can activate both Smad-dependent canonical and Smad-independent non-canonical signalling cascades. The TGF-β activated TGFBR1 phosphorylates the Smad2/3 complex which then associates Smad4, before translocating to the nucleus to regulate the transcription of different targeted genes involved in tumour suppression during tumorigenesis (e.g., *p15^INK4b^* and *p21^Cip1^*), as well as tumour progression (e.g., *Snail* and *Vimentin*). The Smad-dependent TGF-β canonical signalling pathway can also be antagonised by Smad7 through inhibiting the binding of the Smad2/3 complex with Smad4. In the Smad-independent non-canonical signalling cascades, the activated TGFBR1/2 receptors induce downstream signalling through the Ras/Raf/MAPKs (JNK/p38/ERK), PI3K/AKT, MAP3K7(TAK1)/NF-κB, and Rho family of small GTPase-dependent signalling pathways. The TGF-β activated JNK/p38/ERK-MAPKs also crosstalk with Smad2/3/4 to modulate downstream signalling to influence cancer development. The Rho family of small GTPase-dependent signalling pathways (e.g., Rho/ROCK) are involved in epithelial–mesenchymal transition (EMT). The activated PI3K/AKT pathway induces mTOR to promote cell proliferation and EMT. The TGF-β signals also activate NF-κB signalling to modulate inflammatory response.

TGF-β executes its functions through canonical and non-canonical pathways (Figure 1). In the canonical TGF-β/Smad pathway, TGF-β ligands bind to a heterotetrameric TGF-β receptor complex, composed of dimers of type I (TGFBR1) and type II (TGFBR2) TGF-β receptors. The ligated TGF-β and receptor complexes then become phosphorylated and activated before recruiting and phosphorylating Smads, including Smad2 and Smad3. Smad2/3 phosphorylation induces the subsequent recruitment of Smad4 to the Smad2/3 complex which then translocates to the nucleus. Smad3 is the transcription factor (TF) and the component of the complex that directly binds to DNA, and the Smad complexes then cooperate with other cofactors to regulate multiple downstream mechanisms through directing transcriptional activation and suppression [5]. For example, TGF-β mediates the transcriptional regulation of effector genes including the cyclin-dependent kinase (CDK) inhibitors p15^INK4^ [6] and p21^Cip1^ [7] to modulate cell cycle arrest. Besides, the TGF-β/Smad axis also promotes the stemness and epithelial–mesenchymal transition (EMT) of cancer cells by restoring mesenchymal phenotypes and upregulating the expression of genes, such as *Snail* and *Vim* [8,9]. Meanwhile, the TGF-β/Smad pathway also has a negative feedback mechanism mediated through Smad7 competitive binding to TGFBR1 and blocking the TGF-β/Smad pathway signalling [10]. For the non-canonical TGF-β pathway, the activated TGF-β crosstalks with other signalling pathways, such as Rho, phosphoinositide 3-kinase (PI3K), and mitogen-activated protein kinase (MAPK) signalling cascades, to promote EMT [11], cancer invasion [12], and angiogenesis [13]. In consequence, both the canonical and non-canonical TGF-β pathways play an important role in cancer progression [14].

Accumulating evidence has indicated that TGF-β has a dual function in cancer progression, with the different TGF-β signalling pathways switching between the two phenotypes of tumour suppression and tumour promotion [15]. More importantly, the role of TGF-β in the immunosuppressive TME and immune cells has attracted increasing attention. In this review, we will evaluate the complex role of TGF-β in cancer progression, focusing primarily on its function in the regulation of immune responses and TME development.

## 2. TGF-β in Cancer Initiation and Progression

### 2.1. Role of TGF-β as Tumour Suppressor

TGF-β plays multiple roles in the early stages of carcinogenesis, one of which is to modulate cell proliferation arrest. For example, TGF-β can induce cell cycle arrest via the cAMP response element-binding protein (CREB) to mediate histone acetylation and transcriptional activation of plasminogen activator inhibitor type-1 in a p53/Smad-dependent manner to maintain the anti-proliferative effects to suppress cancer tumorigenesis [16]. Besides, TGF-β can also activate the forkhead box O1 (FOXO1) transcription factor to induce the expression of the cyclin-dependent kinase inhibitor p21^Cip1^ at the transcriptional level to enforce a G0/G1 phase cell cycle arrest. This is mediated via the TGF-β/FOXO1/p21^Cip1^ axis to keep cells in a quiescent or senescent state [17]. TGF-β can also promote normal cell differentiation as an indirect tumour suppressive antiproliferative mechanism by promoting the activity of the stress-activated p38MAPK signalling pathway [18]. In agreement, haematopoietic stem and progenitor cells (HSPCs) expressing high levels of active TGF-β1 protein and p38MAPK activity lose their haematopoietic stem cell (HSC) self-renewal and multi-lineage capacity and differentiate into progenitor cells [18]. 

Mechanistically, the role of TGF-β as a tumour suppressor during early tumorigenesis is mediated primarily through its function to cause cell cycle and proliferative arrest, to induce differentiation and apoptosis, and to block paracrine factor production [17]. The anti-proliferative tumour suppressive function of TGF-β is mediated primarily through the induction of CDK inhibitor expression and the suppression of c-Myc expression, at the transcriptional level, to arrest cell cycle progression at the G1 and S phases of the cell cycle. For example, in normal epithelial cells, TGF-β induces the expression of the cyclin-dependent kinase inhibitor (CKI) p15^INK4b^, which competes with cyclin Ds for the formation of cyclin D complexes with CDK4/6, and of p21^Cip1^, which inhibits the activity of the cyclin E/A-CDK2 complexes directly. Specifically, the Smad3/4 complexes associate and cooperate with FOXO transcription factors to transcriptionally activate the promoters of the *CDKN2B* gene, which encodes p15^INK4b^, and of *CDKN1A*, which encodes p21^Cip1^. The induced CKI p15^INK4b^ can also displace p27^Kip1^ from the cyclin D-CDK4/6 complexes, releasing the Cip/Kip CKI (e.g., p21^Cip1^, p27^Kip1^, and p57^Kip2^) to inhibit the cyclin E/A-CDK2 complexes to restrict cell cycle progression through late G1 and S phases of the cell cycle. Indeed, TGF-β has also been shown to stimulate the expression of CKIs, such as p15^INK4b^, p21^Cip1^, p27^Kip1^, and p57^Kip2^, in a cell type- and context-dependent manner. For example, TGF-β induces the expression of p21^CIP1^ in T cells, p57^Kip2^ in haematopoietic stem/progenitor cells, and p15^INK4b^ and p21^Cip1^ in astrocytes, neural progenitor cells, and epithelial cells to cause cell cycle and proliferation arrest. The TGF-β-induced Smad3/4-containing protein complexes also repress the expression of transcription of c-Myc, a potent oncogene which plays a pivotal role in cell cycle entry and proliferation. Besides cell cycle progression and proliferation, TGF-β also limits proliferation indirectly through promoting differentiation. For instance, TGF-β signalling also represses the expression of Id proteins (inhibitor of differentiation/DNA binding), which inhibit some key differentiation pathways. In addition, TGF-β signalling can also trigger apoptosis, programmed cell death to restrict cell proliferation. Consistently, TGF-β can promote apoptosis through inducing the expression of modulators of programmed cell death, including the death-associated protein kinase (DAPK), the growth arrest and DNA damage 45β factor (GADD45β), the death receptor FAS, etc., and often in a cell type-dependent manner. Lastly, TGF-β can also restrict epithelial cell proliferation and tumorigenesis by inhibiting the production of mitotic growth factors, such as hepatocyte growth factor (HGF), by the fibroblasts and immune cells in the local TME. Together, these findings suggest that TGF-β targets different gene targets via discrete signalling pathways in distinct cell types and settings to function as a pleiotropic suppressor of cancer onset [17].

### 2.2. Role of TGF-β as Tumour Progression Promoter

Conversely, TGF-β can also function as an oncogene and promote cancer cell progression by activating the PI3K/AKT/mTOR pathway [19]. Appropriately, TGF-β is associated with poor prognosis and cancer progression in osteosarcoma [19]. Alternatively, TGF-β1 has also been shown to activate Golgi membrane protein 1 (GOLM1; also called GP73) through lipid rafts to suppress Smad-dependent tumour-suppressive signals and, at the same time, induce the extracellular signal-regulated kinase (ERK) MAPK signalling pathway to promote tumorigenesis and progression in liver cancer [20]. In addition, TGF-β also regulates tissue infiltration and migration by enhancing bFGF signalling through the FGFR/FRS2/ERK axis in paediatric medulloblastoma [21]. The importance of MAPK in mediating the oncogenic function of TGF-β is exemplified by the fact that the Smad4 phosphatase Wip1 can restrain the TGF-β-induced cell growth arrest, migration, and invasion and enhances the tumorigenicity of cancer cells by repressing Smad4 activity via antagonising MAPK function. Mechanistically, Wip1 selectively dephosphorylates Smad4 at a specific MAPK phosphorylation site to inhibit its nuclear accumulation and stability [22].

Increasing evidence has indicated that TGF-β can aggravate EMT, a key process involved in metastasis during cancer progression. For example, the TGF-β/Smad pathways can promote EMT and contribute to lung cancer progression by driving the expression of the DNA binding protein family member ID1 and EMT-related transcriptional factor Snail. Besides that, TGF-β can also induce EMT via the PI3K/AKT axis in a tuberous sclerosis protein complex-dependent manner [23]. Furthermore, TGF-β also promotes EMT and enhances cancer cell invasion and migration via activating Rho and Rho-associated protein kinases (ROCKs), whereas the malignant phenotypes induced by TGF-β via the Rho/ROCK signalling pathway can be blocked by miR-335-5p and farnesyl pyrophosphate synthase inhibitors in non-small cell lung cancer (NSCLC) [24,25]. The activation of MAPKs and downstream genes is another crucial mechanism by which TGF-β mediates cellular responses. TGF-β induces Ras and recruits Raf to the plasma membrane. The TGF-β-activated Ras and Raf then transmit signals via MAP kinase kinases (MKKs) to MAPKs to promote EMT. Cho et al. have found that the activated TGF-β signals induce the degradation of downstream Raf kinase inhibitory protein and promote the activation of the MAPK signalling pathway, which in turn results in the transcriptional suppression of p53 to facilitate EMT [26]. Moreover, the TGF-β pathway can also crosstalk with NF-κB to increase the expression of Twist to enhance EMT, and this cooperation between TGF-β and NF-κB pathways is again mediated by MAP kinase kinase kinase 7 (MAP3K7), an upstream MAPK family member [27,28]. Recently, CCAAT enhancer-binding protein alpha (C/EBPα) has been identified as a “gatekeeper” gene in TGF-β-induced EMT. More specifically, the TGF-β-induced downregulation of C/EBPα allows epithelial cells to undergo malignant transformation of breast epithelial cells, while abundant C/EBPα expression can effectively prevent breast cancer tumourigenesis [29].

Nevertheless, there is also evidence that TGF-β can also suppress tumour progression by generating a lethal EMT. Specifically, David et al. have found that TGF-β induces the expression of SRY-box transcription factor 4 (SOX4) to promote pro-apoptotic events via Smad4-mediated repression of kruppel-like factor 5 (KLF5). This in turn induces the lethal effects and phenotype transformation of EMT in TGF-β-sensitive pancreatic ductal adenocarcinoma (PDA) cells [30]. In agreement, the pro-apoptotic regulator WT1 is another gene that has been reported to be induced during TGF-β/Smad4-derived lethal EMT [31].

### 2.3. Role of TGF-β in Normal and Cancerous Cells

Overall, these findings suggest that TGF-β plays distinct and sometimes contrasting roles during cancer initiation and development. In general, in non-cancerous cells, TGF-β normally functions as a tumour suppressor to limit cell proliferation and promote apoptosis in order to exert growth inhibition during early stages of carcinogenesis. During later stages of cancer development, TGF-β often switches to a cancer promoting role, which is essential for tumour progression and metastasis. In concordance with this conjecture, one transcription factor that plays a key role during this TGF-β functional switch is distal-less homeobox 2 (Dlx2) [32]. In normal mammary epithelial cells, Dlx2 neutralises the TGFβ-induced cell cycle arrest and apoptosis by multiple mechanisms. Essentially, Dlx2 functions as a transcriptional repressor of TGFBR2 gene expression, restricting the downstream canonical Smad-dependent TGF-β signalling and expression of the cell cycle inhibitors, such as p21^Cip1^, and enhancing the expression of the potent mitogenic transcription factor c-Myc. Conversely, Dlx2 can also directly drive the expression of the epidermal growth factor (EGF) family member betacellulin to promote cell survival by stimulating EGF receptor (EGFR) signalling. Moreover, Dlx2 also further supports tumour growth and metastasis. These results establish Dlx2 as a critical switch in shifting TGF-β from its tumour-suppressive functions in early tumorigenesis to its tumour-promoting functions during cancer progression [32]. 

### 2.4. Regulation and Role of TGF-β in the Tumour Microenvironment

The TME provides the structures and materials for tumour maintenance and progression, and comprises the cellular and extracellular materials surrounding the tumour mass. Essentially, TME serves as a platform for cancer cells to reprogramme infiltrating stromal cells, thereby promoting tumorigenesis, as well as cancer invasion, metastasis, and resistance to therapy. TGF-β is secreted in a latent form as a large latent complex (LLC) which is primarily embedded in extracellular matrix (ECM) and is partially anchored on the cell surface [33,34]. It will remain as an inactivated complex until it is processed further to release the active TGF-β. Within the TME, a myriad of cell types can produce TGF-β, and the generation and processing of this important cytokine is mediated through diverse and complex mechanisms. For example, TGF-β produced by epithelial cells is induced via matrix metalloprotease (MMP) stimulation and EGFR activation [35]. In addition, TGF-β secretion in macrophages is regulated by the Notch signalling pathways [36]. Moreover, signal transducer and activator of transcription 6 (STAT6) and Furin can also cooperate to induce the production of TGF-β by T lymphocytes [37]. Furthermore, inactive forms of TGF-β can be stimulated and released by integrin-mediated activation, protease cleavage, and latency-associated peptide (LAP) dissociation upon X-ray irradiation [38,39,40]. It is also worth noting that a recent study has suggested a novel integrin-activated form of TGF-β that does not require release from the latent TGF-β complex [41]. Together, these observations illustrate the complexity and diversity of the mechanisms by which TGF-β are generated and activated. Moreover, diverse cell types are involved in modulating the effects of TGF-β on the development and the reprogramming of the TME, as well as during cancer progression. For example, migratory dendritic cells (DCs) can activate TGF-β via an integrin-dependent manner and promote naïve CD8+ T cell differentiation into epithelial resident memory T cells (eTRM) [42]. Notably, the interactions between non-immune cells and immune cells are also essential for TGF-β activation and function. It has been found that keratinocytes help to preserve epithelial-resident DCs and eTRM by inducing integrin expression and thereby TGF-β activation [43]. Besides that, tumour-initiating cells also release IL-33 to induce macrophage differentiation, which ultimately promotes TGF-β secretion and signalling to cancer stem cells (CSCs) to create a CSC niche for the maintenance of a stem cell pool within the TME [44]. In concordance, Takasaka et al. have also observed that integrin αvβ8 is highly expressed on the cancer cell surface and that the integrin αvβ8-expressing tumour cells can evade host immunity by regulating TGF-β activation in immune cells [45]. On the contrary, integrin β1-mediated TGF-β activation may also drive tumor suppression. In a human melanoma xenograft model, cell surface integrin β1-activation can increase TGF-β activity, which culminates in stromal activation and angiogenesis but also an accumulation of intra-tumoral CD8+ T cell infiltration within the TME. The latter recruitment of CD8+ T lymphocytes can result in an attenuation of tumour growth and long-term survival, suggesting a role of TGF-β in immune surveillance against tumours [46]. 

Besides targeting cancer cells, TGF-β also exerts its effect on stromal cells of the TME through controlling their differentiation, angiogenesis, and metabolic reprogramming during tumourigenesis and cancer progression. For example, amphiregulin expressed by macrophages can induce TGF-β activation to mediate pericyte differentiation into myofibroblasts to direct tissue restoration during inflammation [47]. In addition, TGF-β-licensed mesenchymal stromal cells (TGF-β MSCs) can contribute to immunosuppression by altering the phenotypes of macrophages and by promoting regulatory T cell (Treg) expansion [48]. Previous studies have also showed a strong association between stromal TGF-β levels and poor prognosis in hepatocellular carcinoma (HCC), as well as colorectal cancer (CRC), suggesting an important role for stromal TGF-β in promoting an immunosuppressive TME [49,50]. Moreover, TGF-β is able to stimulate myofibroblasts and other stromal cells to boost the synthesis of collagen crosslinked enzymes, especially lysyl oxidases (LOs) and matrix metallopeptidases (MMPs), to improve collagen crosslinking during the early stages of carcinogenesis [51]. Collagen crosslinking mediated by the lysyl oxidase family of enzymes (LOX, LOXL1-4) contributes to the pathogenesis of idiopathic pulmonary fibrosis (IPF), a progressive scarring lung disease which predisposes patients to lung cancer, mostly NSCLC. A previous study has also shown that the novel lysyl hydroxylase 3 receptor recruits MMP9 to the surface of fibroblasts and activated TGF-β and actin alpha 2 (α-SMA) functions during fibroblast differentiation [52]. At the same time, TGF-β can also downregulate the synthesis and expression of MMP family members, such as MMP2, MMP7, and MMP8, to facilitate the regulation of the ECM [53]. In addition, increased matrix protein synthesis and reduced matrix proteinase activity due to increased TGF-β activity can also contribute to the tumour ECM remodelling and result in desmoplasia, which is commonly found in many types of tumours, particularly pancreatic and renal cell carcinomas, as well as sarcomas [54]. In agreement, bone marrow-derived mesenchymal stem cells (BM-MSCs) have been shown to inhibit the TGF-β/SMAD pathway to repress hypoxia-inducible factor 1 subunit alpha (HIF-1α) and VEGF protein expression in the ECM to restrict liver fibrosis and early HCC tumourigenesis [55]. Similarly, pharmaceutical inhibition of the TGF-β signalling can promote the epithelial differentiation of human adipose-derived mesenchymal stem cells (ADSCs) during EMT through the downregulation of mesenchymal genes (e.g., Slug, zinc finger E-box binding homeobox 1 (ZEB1), integrin α5 (ITGA5), and vimentin (VIM)) and upregulation of epithelial genes (e.g., E-cadherin, epithelial cell adhesion molecule (EpCAM), zonula occludens-1 (ZO-1), occludin, deltaN p63 (δNp63), transcription factor 4 (TCF4), and Twist family bHLH transcription factor (TWIST)) to reverse EMT [56]. Moreover, cancer-associated fibroblasts (CAFs) are an abundant and active population of stromal cells and play a major role in conditioning the TME. TGF-β1 can drive the conversion of normal fibroblasts to CAFs in the TME. For example, CAFs have a key role in the tumourigenesis of oral squamous cell carcinoma (OSCC), the most common cancer of the oral cavity. In this context, TGF-β can induce the conversion of normal oral fibroblasts (NOFs) into CAFs, which are fibronectin type III domain-containing 1 (FNDC1), serpin peptidase inhibitor type 1 (SERPINE1), stanniocalcin 2 (STC2), and type I collagen, to promote the proliferation and invasion of OSCC cells, resulting in a more aggressive tumour phenotypes [57]. The transformation of fibroblasts, which is promoted by the TGF-β/Smad pathway, is also involved in the process of pulmonary fibrosis and early lung cancer tumourigenesis. Consistently, it has been shown that TGF-β1 can induce lung fibroblast transition to myofibroblasts and the expression of α-SMA via the Rho/ROCK and TGF-β/Smad pathways in lung fibroblast–myofibroblast transformation [58]. Furthermore, CAFs also interact with immune cells in the TME, including macrophages, mast cells, natural killer (NK) cells, DCs, myeloid-derived suppressor cells (MDSCs), tumour-associated neutrophils (TANs), and T lymphocytes, to promote tumour progression and immune evasion [59,60]. For example, CAFs and tumour-associated macrophages (TAMs) have been shown to cooperate in driving neuroblastoma progression and invasion [59]. Similarly, CAFs from the HCC TME can enhance the generation of regulatory DCs, to facilitate tumour progression and immune evasion through the production of high levels of TGF-β, impairment of T cell proliferation, and expansion of the Treg population [59]. In addition, the activation of TGF-β signalling in CAFs has been linked to immunosuppression mediated through the induction of a unique set of ECM genes. Moreover, the immunosuppression triggered by TGF-β signalling in CAFs is caused by immune checkpoint blockade and has been shown to be able to lead to anticancer immunotherapy failure in melanoma and bladder cancer [61]. Together, these findings provide solid evidence that TGF-β plays an unparalleled role in mediating tumour growth, metastasis, and chemoresistance and immune evasion through the TME and is therefore a promising target for anticancer intervention.

Besides deregulation of TGF-β expression and activity itself, changes in the expression of components of the TGF-β downstream signalling pathways can also modulate tumourigenesis, cancer progression, and chemoresistance. DC-STAMP domain-containing 1-antisense 1 (DCST1-AS1) and annexin A1 are two genes functioning downstream of TGF-β that play key roles in EMT and cancer chemotherapy resistance. DCST1-AS1 regulates the expression of EMT-related genes including MMP2, MMP9, and E-cadherin. DCST1-AS1 has been found to bind to annexin A1 directly in triple-negative breast cancer (TNBC) cells to modulate the transcription of its downstream regulatory genes involved in EMT and cancer drug resistance [62]. In addition, the TGF-β/MAPK pathway also participates in the transcriptional activation of ETS1 to modulate TGF-β-mediated chemoresistance in liver cancer [63]. Conversely, Ma et al. have found that DNA methyltransferase inhibitor treatment can restore the decreased expression of TGFBR2 caused by hypermethylation on the gene promoter, and this treatment can successfully promote cell cycle arrest and inhibit cancer proliferation in esophageal squamous cell carcinoma (ESCC) [64]. Moreover, the epigenetic silencing of the TGF-β gene, *TGFB1*, also contributes to trastuzumab resistance in Her2+ breast cancer [65]. Another point worth mentioning is the function of non-coding RNAs in TGF-β pathway regulation and their effects on tumourigenesis. The microRNA miR-495 is one that inhibits the expression of homeobox C6 and the function of TGF-β1. Its overexpression in CSCs contributes to EMT reversion, apoptosis, and the decreased proliferation and migration of CSCs [66]. The miRNA-200 family is another important group of microRNAs that promote cyst formation and ovarian cancer spread through targeting TGF-β expression [67]. Moreover, some recent studies show that circular RNAs, such as circCACTIN and circCCDC66, also affect cancer progression by regulating the TGF-β pathway [68,69], and circular RNA cESRP1 can increase the chemosensitivity of cancer cells by inducing Smad7 and blocking the TGF-β pathway [70].

## 3. TGF-β in Immune Surveillance

**Figure 2:**
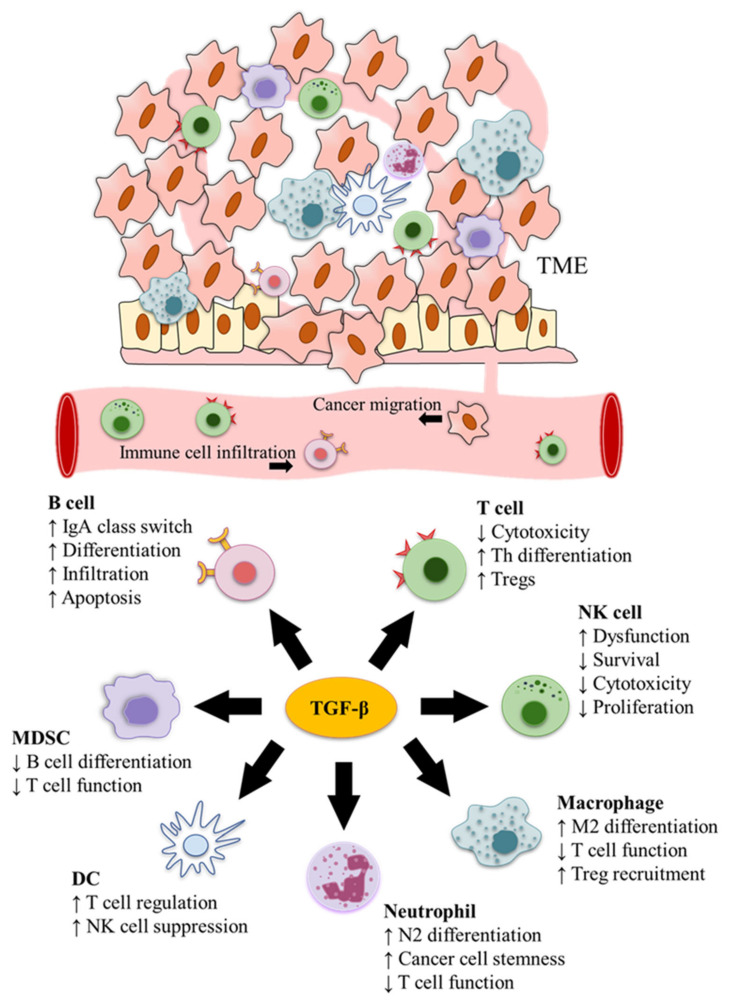
TGF-β functions in cancer immune surveillance. TGF-β modulates cancer immunity and has pivotal roles in the immunomodulation of the tumour microenvironment (TME) and different immune cells.

TGF-β is produced not only by cancer cells but also different types of immune cells in the TME [71]. Accordingly, based on immunohistochemistry staining, Ohtani et al. have observed that the latent form of TGF-β1 is mainly localised in immune cells instead of cancer cells, with macrophages, DCs, and T cells being the predominant sources of latent TGF-β1 in cancer stroma [71]. In fact, both the autocrine and the paracrine pathways play an important role in mediating the effects of TGF-β on tumour growth and progression [72]. For example, autocrine TGF-β1 in T cells promotes Th17 cell differentiation [73], and many types of immune cells, including Tregs and macrophages, can perform a similar function by releasing TGF-β1 anchored on the cell surface to prime paracrine signalling [74]. In consequence, TGF-β plays a pivotal role in the immunomodulation of TME and different immune cells (Figure 2). The main functions of TGF-β in cancer immunity are listed in Table 1.

### 3.1. T Cells

TGF-β affects the survival, activation, and differentiation of different lineages of T cells. These effects are not only caused by TGF-β-induced cell cycle arrest and differentiation in CD4+ and CD8+ T cells directly [101], but the TGF-β-stimulated stromal cells can also affect T cell functions. For example, MSCs can inhibit the activation of T cells by enhancing the expression of latent TGF-β1 complexes on the cell surface [102]. In bone metastasis of castration-resistant prostate cancer, increased TGF-β levels also prevent Th1 lineage development. Combining the TGF-β1 blockade with immune checkpoint blockade therapy can effectively reverse the immunosuppressive state by increasing the number of Th1 and CD8+ T cells to achieve significant tumour regression and improve patient survival [75]. In addition, the blockade of different isoforms of TGF-β, including TGF-β1 and TGF-β2, has been shown to enhance tumour immunity through increasing the immune response from the Th1 population and the production of interferon gamma (IFN-γ), which is more efficient under programmed cell death 1 (PD-1) blockade [76]. It is worth noting that combining TGF-β with other cytokines may stimulate cytotoxic T cell differentiation to produce even more powerful anti-tumour functions. Specifically, IL-4 and TGF-β have been reported to be indispensable for the cell priming and differentiation of IL-9-producing CD4+ Th9 cells, which are a subset of CD4+ T helper cells with a powerful anti-tumour capacity [77]. Moreover, TGF-β can directly promote the differentiation of T helper 17 (Th17) cells to drive cancer progression [103]. Specifically, TGF-β1 increases the population of IL-22-producing Th17 cells via activation of PI3K signalling and thereby promotes tumour growth, aggressiveness, and treatment resistance through the subsequent uncontrolled high levels of IL-22 [78]. In addition, TGF-β signalling also drives the *trans*differentiation of Th17 cells into Foxp3+ regulatory T cells (Tregs), and this process directly affects immune responses and results in immune tolerance and immunosuppression in the TME [104,105]. Accordingly, the induction of Tregs will suppress the development of cytotoxic CD8+ T cells and help tumour cells to escape from immune surveillance [106]. TGF-β also promotes the generation of antigen-specific in vitro-induced Tregs (iTregs) by inducing the expression of Foxp3 directly in the presence of IL2. The TGF-β-induced iTregs suppress the proliferation of naïve effector T cells, inhibit the function of Th17, and prevent antigen presentation by DCs [79]. Indeed, the immunosuppression functions mediated by iTregs have shown great potential in ameliorating autoimmune diseases, such as arthritis and stromal keratitis, by promoting differentiation of Th17 and impairing CD4+ T cell function [107,108]. However, these immunosuppressive properties exerted by iTregs can also interfere with cancer treatment and present a barrier to cancer immunotherapy. In concordance, antigen-specific iTregs have been demonstrated to suppress the immunotherapy effects of cancer vaccines and promote cancer growth and progression [109,110]. Not surprisingly, silencing TGF-β1 is also associated with decreased levels of Tregs and enhanced activation of effector T cells in the TME, which has a synergistic effect with VEGFA depletion using siRNA in cancer immunotherapies [80]. In addition, knocking out other genes downstream of TGF-β signalling also suppresses Tregs and enhances cancer immunity. For example, TGFBR2 knockout chimeric antigen receptor-modified T cells can induce Treg conversion and promote tumour regression in vivo [111]. Increasing Tregs induced by TGF-β signalling in turn increases the expression and activation of TGF-β1 [112].

### 3.2. B Cells

TGF-β can also affect B cell development by regulating the differentiation, proliferation, and apoptosis of B cells [113]. In addition, it is also one of the cytokines that dictate the immunoglobulin IgM to IgA class switching in B cells. In Peyer’s patches, IgA switching requires the interaction between B cells and DCs because the integrin αvβ8 expressed on DCs activates TGF-β to induce B cell maturation and IgA production [114]. In concordance, the B cells located in the vicinity of tumour cells are IgA positive and exert immunosuppressive effects on the TME. In CRC, IgA-producing B cells also express the high levels of programmed death ligand 1 (PD-L1) and TGF-β, which restrict the production and activation of cytotoxic CD8+ T cells and enhance immunosuppression [81]. Besides facilitating the B cell immunoglobulin class switch to IgA, TGF-β/Smad signalling also modulates DNA methylation to block B cell terminal differentiation into plasma cells. In particular, downregulation of TGF-β1 induces the generation of a CD23-negative cell subpopulation during the B cell to plasma cell transition, a process caused by the hydroxymethylation of PR domain 1 (PRDM1), a gene essential for plasma cell fate determination [115]. Additionally, TGF-β1 can inhibit the generation of B lymphoid progenitor colonies by decreasing stromal IL-7 production to limit lymphopoiesis [116], and it inhibits T cell and NK cell proliferation and anti-tumour effects via promoting the function and infiltration of tumour-educated B cells [82]. Moreover, in combination with cyclic adenosine monophosphate, TGF-β1 induces the peripheral blood resting human B cells to undergo apoptosis via a B cell lymphoma 2-independent mechanism [117].

### 3.3. Natural Killer (NK) Cells

**Figure 3:**
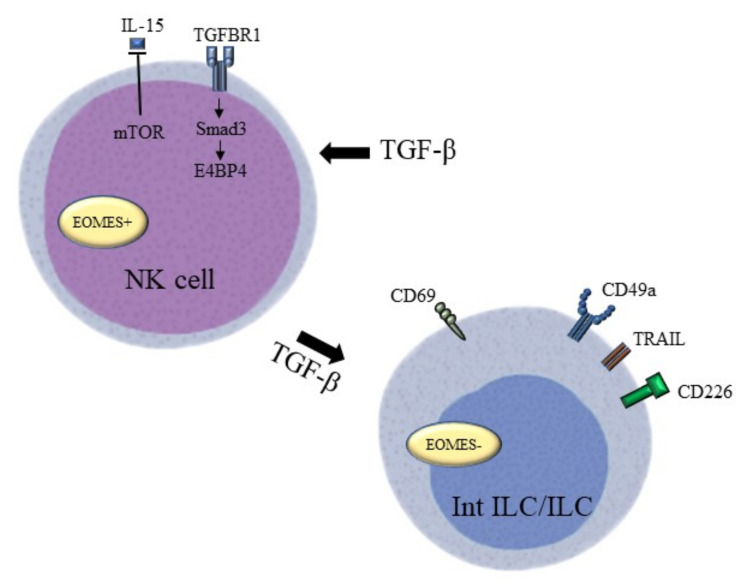
TGF-β targeting factors that “lock” NK cell functions. TGF-β impairs the balance between activating signals and inhibitory signals on NK cells to block the activation of NK cells. Their anti-tumour activity is reduced by downregulating the mTOR pathway and upregulating the Smad3/E4BP4 pathway. The conversion of NK cell to innate lymphoid cell under the high levels of TGF-β leads to the loss of the metastasis-restraining function of NK cells through the expression of CD69, CD49a, and TRAIL, and the loss of EOMES.

NK cells are a major cellular component functional during innate immune responses and they can kill tumour cells without any priming or prior activation. TGF-β has been found to be able to modulate NK cell function and activity in several ways (Figure 3). Mamessier et al. have identified TGF-β as an important stromal factor involved in tumour-induced dysfunction and enhanced anti-tumour immunity of NK cells in breast cancer [118]. Specifically, high levels of circulating TGF-β1 in the peripheral blood of HCC patients can disturb the balance between CD96, CD226, and T cell immunoglobulin and immunoreceptor tyrosine-based inhibitory motifdomain levels in NK cells, which causes NK cell dysfunction. Patients with a higher cumulative percentage of CD96+ NK cells within tumours also exhibit poorer disease-free survival, suggesting that TGF-β1 regulates the cytotoxic potential and cell immunity of NK cells against tumour cells [119]. TGF-β induces the conversion of NK cells into intermediate type 1 innate lymphoid cells (intILCs) in the TME to restrict innate immune surveillance. This type of innate lymphoid cell (ILC)-like phenotype loses the metastasis-restraining function of NK cells through the expression of CD69, CD49a, and TNF superfamily member 10 (TNFSF10), and the loss of the T-box transcription factor Eomesodermin (EOMES) due to constitutively active TGF-β signalling. In support of this observation, NK cell-deficient mice were unable to restrict lung metastasis in the presence of TGF-β-activated ILCs [120]. Moreover, Smad4 can hamstring this cell conversion by restricting the function of the non-canonical TGF-β pathway in NK cells [121]. Different recognition receptors of NK cells can be triggered by adverse environmental conditions in the TME to activate NK cell cytotoxicity towards tumour cells, while TGF-β1 secreted by the tumour cells demonstrated immunosuppressive effects on NK cells. TGF-β1 probably causes the conversion of tumour-associated NK cells to a non-cytotoxic phenotype in prostate cancer by impairing NK cell receptor D (NKG2D) and DNAX accessory molecule-1 (DNAM-1) [83]. Meanwhile, the loss of phenotype in circulating NK cells with activating receptors, including NKp30, NKp46, NKG2D, and DNAM-1, showed a positive association with tumour progression in gastric cancer. As observed in the previous prostate cancer study, TGF-β1 represses the expression of activating receptors. It is encouraging that the addition of TGFBR1 inhibitors can restore the cytotoxic phenotype of NK cells, which highlights the potential of TGFBR1 and the TGF-β pathway in cancer treatment [122]. Furthermore, the blockade of IL-15, a cytokine responsible for the survival and development of NK cells, can also lead to depletion of the activating receptor NKG2D. Equally, TGF-β1 can also suppress the induction of mTOR activity mediated by IL-15 in murine models of melanoma, breast cancer, and prostate carcinoma. In particular, TGF-β1 inhibits the proliferation of recognition receptors, the production of cytotoxicity cytokines, and the anti-metastatic functions of NK cells [84]. In addition, TGF-β1 can also exert a negative effect on NK cell-mediated immune surveillance via the Smad3/E4BP4 axis to suppress NK cell proliferation and their cancer-killing effects. Accordingly, TGF-β1 suppresses E4BP4 expression in NK cells in a Smad3-dependent manner, and depletion of E4BP4 in NK cells significantly promotes tumour growth [85].

### 3.4. Myeloid-Derived Suppressor Cells (MDSCs)

MDSCs play an essential role in tumour immune evasion by regulating the anti-tumour immune responses during cancer progression. In NSCLC, the TGF-β pathway can stimulate the expression of CD39 and CD73 in MDSCs through activation of HIF-1α to inhibit T cell and NK cell function, thus helping cancer cells to avoid immune surveillance [86]. Additionally, TGF-β1 secreted by MDSCs can accelerate tumour growth and increase tumour burden in lung cancer in response to lung microenvironment changes induced by carbon nanomaterial [123]. Wang et al. have also reported a potential mechanism of TGF-β1 in MDSC-derived immunosuppressive function. In this scenario, MDSC-derived TGF-β1 inhibits IL-7 function, blocks signal transducer and activator of transcription 5A (STAT5A) phosphorylation, and impairs B cell differentiation [87]. In addition, MDSCs educated under TGF-β stimulation can suppress CD8+ T lymphocyte cancer-killing effects by secreting nitric oxide synthase 2or arginase 1 [88]. At odds with most previous findings, Jayaraman et al. have also uncovered that although cancer-induced MDSCs play a key role in tumour immune evasion, they can be conditioned with TGF-β1 to acquire a novel immune-stimulatory phenotype, losing the ability to inhibit T cell proliferation and acquiring an enhanced antigen-presenting capability. Thus, these TGF-β1-induced TGFβ-MDSCs show decreased immunosuppressive effects, enhanced antigen-presenting ability, and increased cancer-killing activity [89].

### 3.5. Macrophages

Macrophages are phagocytes that are the first line of defence against pathogenic tissue damage. They have heterogeneous populations and are typically defined as the classically activated M1 macrophages or the alternatively activated M2 macrophages [124]. TAMs are important components of the TME and are strongly associated with poor prognosis in solid tumours, while M2-like TAMs have also been suggested to serve as prominent metastasis promoters with anti-inflammatory functions in the development of TME [125,126].

TGF-β is an important cytokine for TAM function, and it stimulates monocyte recruitment and macrophage differentiation. This cytokine has been shown to inhibit tumour regression by hindering the activation of MHC-II+ TAMs and production of IFN-α. In consequence, the blockade of the TGF-β pathway or its functions may improve IFN-α-mediated cancer therapy [127]. Moreover, TGF-β1 has a well-established role in the M2 phenotype differentiation of TAMs. Zhang et al. have found that TGF-β1 promotes the expression of anti-inflammatory cytokine IL-10 and suppresses the pro-inflammatory cytokines, including tumour necrosis factor alpha (TNF-α) and IL-12, through the TGF-β/Snail signalling pathway [90]. In addition, the expression of TGF-β is also regulated by other pro-inflammatory cytokines, including IL-6 and IL-17, in TAMs [91,92,93]. In previous studies of gastric cancer, IL-6 has been shown to induce the M2 differentiation and increase the expression of TGF-β and IL-10 in TAMs via promoting STAT3 phosphorylation and activation, and this phenomenon augments the proliferation and migration of cancer cells [91]. Similar mechanisms were also found in the progression of NSCLC, where highly expressed plasminogen activator inhibitor 1 (PAI-1) enhances the expression of cytokines, including C-C motif chemokine ligand 17 (CCL17), CCL22, IL-6, and TGF-β1, in TAMs to promote cancer progression [92]. The upregulated TGF-β1 also participates in M2 differentiation of TAMs in glioma in a hypoxia-mediated manner via the AKT/PI3K pathway [128]. Together, these findings underscore the immense therapeutic value of TGF-β1 in cancer immunosuppression.

Additionally, macrophage-secreted TGF-β also affects the development of TME by regulating other immune cells. For example, TGF-β secreted by macrophages can suppress cytotoxicity of T cells in malignant pleural effusion, and TAM-derived TGF-β has the ability to induce the expression of CCL22 and promote the recruitment of Tregs. The TGF-β-activated Tregs can also secrete IL-8 in the TME to stimulate further TGF-β production in a positive feed-forward loop [129]. Interestingly, TAMs can also modulate TGF-β functions and promote cancer progression by secreting pro-tumour exosomal miRNAs. The microRNA miRNA-501-3p is one of the exosomal miRNAs which are involved in PDA formation and metastasis. In these cases, TGF-β functions through inhibiting type III TGF-β receptor (TGFBR3) expression and activating the TGF-β pathway [130].

### 3.6. Dendritic Cells (DCs)

TGF-β also regulates the adaptive immune response through modulating the function of DCs and their interactions with other immune cells, such as Tregs and NK cells. In the TME, tumour-derived TGF-β inhibits IFN-α, TNF-α, and IL-6 production in tumour-associated plasmacytoid dendritic cells (pDCs) to foster their immune tolerance functions [131]. In addition, TGF-β produced by the TME can also alter DC functions. Enhanced TGF-β signalling within the TME upregulates both the immunoregulatory enzyme indoleamine 2,3-dioxygenase (IDO) in pDCs and the CCL22 chemokine in myeloid DCs (mDCs). The TGF-β-mediated changes in these different DC populations in the TME can direct Treg infiltration and the suppression of anti-tumour immunity [94]. In addition, TGF-β1 also induces the expression of PD-L1 and TNFSF18 in DCs to enhance Treg expansion in lung cancer [95]. TGF-β signalling activation in cancer cells may inhibit DC functions and regulate immune tolerance in the TME. For example, Zhong et al. have found that high levels of TGF-β1 in HCC cells suppress the production and function of DCs and augment immune tolerance [96]. Moreover, blocking signal transduction of TGF-β pathways in self-differentiated DCs increases the IFN-γ production in CD3+ T cells and enhances their cancer-killing effects [132]. These results provide important evidence for DC-mediated immune regulation in the TME.

On the other hand, the TGF-β expressed by DCs also has immunomodulatory effects on the TME. Previous studies have shown that CD11b+CD103- tumour-infiltrating DCs expressing high levels of TGF-β1 and IL-23 can induce tumour-promoting FOXP3-CD4+ Tregs in PDA. These IL-10+IL-17+IFN-γ+ Tregs can confer immunotherapy resistance and poor survival in cancer patients [133]. Besides, DCs educate naïve CD8+ T cells for tissue-resident memory through secreting TGF-β1 [42]. DCs also suppress NK cell activities via TGF-β1, lymphotoxin alpha, IL-12 secretion, and STAT3 phosphorylation [134]. All these studies reveal the diverse functions of TGF-β in immunomodulation and highlight the potential of DC-based cancer immunotherapy. In addition to affecting immune cell infiltration and TME development, DCs can also achieve immunomodulation of TGF-β signalling pathways through secreting antigen-specific exosomes. Exosomes derived from α-fetoprotein+ DCs have been used in novel cell-free vaccines in HCC treatment and they have successfully suppressed tumour growth and increased survival in a tumour-bearing mice model through increasing IFN-γ+CD8+ T cells, IFN-γ, and IL-2, and decreasing CD25+FOXP3+ Tregs, as well as IL-10, and TGF-β production [135]. Some recent studies provided new evidence of TGF-β signalling effects on DCs and tumourigenesis. For example, the single-nucleotide polymorphism (SNP) 509C/T on the TGF-β1 gene promoter region can influence the infiltration of DCs at the invasive margin of the tumour, with the T-allele of the TGFB1 -509C/T SNP having a protective factor for the development of CRC. DCs have always been recognised as the key cellular component of the inflammatory TME and the infiltration of tumours by DCs is associated with favourable prognosis and fewer metastases [136].

### 3.7. Neutrophils

Neutrophils represent 60–80% of the circulating leucocytes and play a pivotal role in host defence. They have high mobility and display a strong activation to release cytokines, defensins, and reactive oxygen species into the TME [137]. TGF-β1 derived from platelets stimulates the recruitment of neutrophils to tumours and promotes metastasis at the early stages of cancer in tumour-bearing mice, hinting at a crucial role of TGF-β1 in shaping the neutrophil responses and the related tumour biology that promotes metastasis [138]. Fridlender et al. have uncovered that tumour-associated neutrophils (TANs) are programmed to induce cell populations with either an anti-tumourigenic N1 or pro-tumourigenic N2 phenotype, and that the TGF-β pathway also regulates this type of polarisation. In the TME, N2 neutrophils are found to have a TGF-β-activated phenotype that releases pro-tumour chemokines and proteases, including CCL2, CCL5, neutrophil elastase, and cathepsin G, that support immunosuppression. By contrast, TGF-β also suppresses N1 neutrophils that express anti-tumour chemokines, modulate arginase reduction, and provide cancer-killing activities [139]. By characterising the development of neutrophil populations into mature high-density neutrophils, mature low-density neutrophils, and immature low-density neutrophils in the TME, Sagiv et al. have further demonstrated that TGF-β treatment forces the N1-like high-density neutrophils to converse into N2-like low-density neutrophils. This conversion has been proven to result in a subsequent positive association between TANs and tumour burden [140]. Neutrophils found in various cancer types, including breast, lung, and colorectal cancer, are often associated with unfavourable patient outcomes [141,142]. Haider et al., have revealed that the high degree of neutrophil recruitment and the poor survival rate at the advanced stage of HCC are correlated with TGF-β1 stimulation. Functional and mechanical analyses in HCC models have also shown that long-term TGF-βl exposure can promote C-X-C motif chemokine ligand 5 (CXCL5) expression to induce a high frequency of neutrophil recruitment [97]. Once the neutrophil numbers increase, the oncogenic hepatocytes can further raise the activity of the TGF-β pathway to maintain the neutrophil recruitment process [98]. Besides that, TANs can also secret TGF-β2 to trigger miR-301-3p-related stem cell characteristics in the oncogenic hepatocytes, which will then secrete a higher level of CXCL5 and recruit more neutrophils to infiltrate the tumour and generate a pro-tumour TME. These interactions between TGF-β2 and neutrophils in the TME form a positive feedback loop to suppress anti-tumour gene expression and promote angiogenesis in HCC [143]. In addition, the TGF-β2-activated neutrophils can further facilitate tumour proliferation by modulating the immune system to lead to differentiation and response changes in other immune cells. It has been proposed that the TGF-β signalling through TGFBR1 in neutrophils participates in the production of TGF-β2 to trigger immunosuppressive activities that can attenuate the T cell response and thereby create a metastatic niche in CRC [99]. Both mouse tumour neutrophils and CRC patient-derived neutrophils have been shown to be able to further inhibit T cell activation by releasing TGF-β1 under high levels of MMP9 within the TME [100]. Collectively, these results suggest that tumour progression in the TME involves the recruitment, differentiation, and activation of neutrophils that are tightly controlled by TGF-β.

## 4. Therapeutic Implications

Cancers develop adaptive mechanisms to suppress the host immune system to facilitate tumour growth and progression. Incidentally, these cancer-evolved immunosuppressive mechanisms are also involved in cancer immunotherapy resistance. Cancers frequently express TGF-β, which drives immune dysfunction in the TME by promoting Tregs and inhibiting the cytotoxic CD8+ and helper Th1 T cells. As TGF-β contributes to immunosuppression and immunotherapy resistance, it is therefore a pivotal target for cancer immunotherapy (Table 2). Based on one of the more substantial pan-cancer transcriptional analyses of ECM gene dysregulation, TGF-β activation in CAFs is a crucial indicator for ECM dysfunction in cancer and this CAF-driven ECM dysfunction is identified as a single important event responsible for the failure of PD-1 blockade cancer immunotherapy [61]. In addition, TGF-β has also been shown to induce PD-L1 expression in cancer cells to further hinder the immunotherapy effects from PD-L1 blockade treatment [144]. As a result, TGF-β inhibition is a promising strategy for overcoming immune checkpoint blockade therapy resistance. Current therapeutic approaches for blocking TGF-β signalling in cancer immunotherapy involve antibodies, proteins, peptides, small molecule inhibitors, and some natural compounds.

As a direct TGF-β-targeting agent, the high-affinity artificial TGF-β1 antibody SRK-181 has been employed successfully in anti-PD-1 cancer treatments to override immunotherapy resistance. Equally, the co-administration of SRK-181 and anti-PD-1 has also demonstrated increased anti-tumour effects and survival benefits in bladder cancer-bearing mice by increasing infiltering CD8+ T cells and decreasing immunosuppressive myeloid cells [145]. Another chemical TGF-β inhibitor, tranilast, which was originally developed as an antiallergic drug, has also demonstrated proven anti-fibrosis and anti-tumour effects. Panagi et al. have shown that combining tranilast and the cytotoxic chemotherapy drug Doxil significantly inhibits tumour progression and improves the efficacy of anti-PD-1 and anti-cytotoxic T lymphocyte-associated protein 4 (CTLA-4) in TNBC [146]. Besides, immunotherapy using antibodies targeting PD-1, TIM-3, and LAG-3 in combination with anti-IL-6 and -TGF-β1 antibodies can enhance treatment outcomes of the E7-TM mRNA vaccine against SOX2 in metastatic lung cancer [147]. Furthermore, a recent study in a lung tumour-bearing mouse model has also shown that fusing a tumour antigen peptide to annexin A5 (AnxA5) significantly enhances its immunogenicity and anti-tumour efficacy when administered after chemotherapy. In this context, cisplatin treatment can activate TGF-β to create an immunosuppressive TME and annexin A5 works as an immune checkpoint inhibitor to reprogramme the TME and restore tumour sensitivity by blocking the expression of TGF-β3 [148]. To circumvent the resistance to checkpoint blockade immunotherapy and target multiple cancer specific pathways concurrently, multifunctional fusion proteins have also been designed and developed as novel cancer immunotherapeutic strategies. Bintrafusp alfa, also known as M7824, is a fusion protein that contains the extracellular domain of TGFBR2 fused to an IgG1 antibody of PD-L1, which traps TGF-β and targets PD-L1 at the same time [149]. This antibody–drug conjugate (ADC) designed to simultaneously target the two immunosuppressive pathways of TGF-β and PD-L1 has shown some encouraging results in some early clinical studies. In an ongoing phase I clinical trial (NCT02517398), bintrafusp alfa has demonstrated good clinical response, safety, and tolerance in advanced head and neck squamous cell carcinoma (HNSCC) that progressed after platinum therapy [150]. In addition, a joint targeting of CXCR1/2, TGF-β1, and PD-L1 using the CXCR1/2 inhibitor SX-682 and bintrafusp alfa also reduces EMT, enhances the infiltration of effector T cells and promotes the blockade of granulocytic myeloid cells in breast and lung cancer mouse models in vivo [151]. Similarly, bifunctional fusion antibodies that simultaneously target the immune checkpoint and disable TGF-β signalling can significantly enhance the efficacy of cancer immunotherapy [152]. Accordingly, compared with traditional CTLA-4 antibody (ipilimumab)- and PD-L1 antibody (atezolizumab or avelumab)-mediated immunotherapy, bifunctional antibody–ligand traps comprising an antibody targeting CTLA-4 or PD-L1 fused to a TGFBR2 ectodomain sequence are more effective in reducing tumour-infiltrating Tregs and inhibiting tumour progression in the TME [152]. Overall, these multifunctional TGF-β pathway-targeting fusion proteins exhibit a good efficacy and manageable safety profile in patients with a range of advanced solid tumours and can therefore have the potential to be developed into effective cancer immunotherapeutic agents [153,154].

Furthermore, due to the immunosuppressive effects of TGF-β on immune cells, targeting TGF-β can restore anti-tumour activity of immune cells. For instance, galunisertib, an inhibitor of TGFBR1, blocks the function TGF-β signalling and enhances the anti-tumour effects of CD133 and Her2 chimeric antigen receptor (CAR) T cells against both glioma and breast cancer [155]. Moreover, SD-208, a novel inhibitor of TGFBR1, induces the infiltration of immune cells, including macrophages, CD8+ T cells, and NK cells, to enhance the immunogenicity of glioma cells and inhibit tumour progression [156]. Furthermore, SD-208 also increases the viability of CD4+ and CD8+ ROR1-specific CAR T cells to enhance the cancer-killing effects [157]. On the other hand, TGFBR1 inhibitor SB431542 is able to decrease tumour burden by eliminating the immune suppression induced by Tregs and regulatory B cells (Bregs) and restoring normal T cell function [158]. For NK cells, the immunosuppressive effects of TGF-β1 on E4BP4 and its target IFN-γ in natural killer NK-92 cells can be reversed via Smad3 silencing in xenograft mouse models of human hepatoma and melanoma [159]. Consistently, the expression of a chimeric receptor with TGFBR2 extracellular and transmembrane domains and the intracellular domain of NKG2D can also neutralise the high level TGF-β-derived immunosuppressive TME and restore higher killing ability and IFN-γ production capacity in the transferred NK cells. In this context, the hybrid receptor converts the TGF-β1 suppressive signals into activating signals. In further agreement, other studies have shown that the anti-tumour effect of TGF-β1 is associated with TGFBR2 activity and is mediated by regulating the cytotoxicity of NK cells, as tumours grow irrespective of TGFBR2 inhibition in mice that lack NK cells [160]. Moreover, the TGF-β superfamily member activin-A can also weaken human peripheral NK cell cytokine production through inhibitory signalling through the type I and II activin receptors, whereas the blockade of activin-A efficiently downregulates cNK-ILC1-like cell differentiation in the TME by downregulating CD49a and CD69, and upregulating EOMES expression in human NK cells simultaneously [161]. Additionally, promoting the differentiation of tumour stem cells and inhibiting proliferation by regulating the TGF-β pathway also provide feasible cancer treatments. For example, RGFP966, an inhibitor of histone deacetylase 3, can block the TGF-β pathway via negative feedback of Smad7, and induce glioma stem cell differentiation into astrocytes [162]. Meanwhile, both pirfenidone and cabazitaxel can achieve effective inhibition of cancer cell proliferation and viability by targeting the TGF-β pathway [163,164].

In addition, several chemicals and natural compounds have also been demonstrated to facilitate TGF-β1-based cancer immunotherapy. For example, retinoic acid can strengthen the anti-tumour effects of kartogenin in the blockage of the TGF-β pathway and inhibition of the viability of retinoid-resistant tumour cells [165]. Furthermore, Lin et al. have found that chidamide can also attenuate the repression of E-cadherin expression caused by TGF-β, and arrest both EMT and cell migration in lung cancer [166]. Similarly, vanadium can also restrict EMT by regulating the TGF-β pathway. Accordingly, the combination of vanadium and the traditional chemotherapy drug carboplatin induces a more potent G0/G1 cell cycle arrest [167]. Similarly, astragaloside IV also achieves cancer treatment via TGF-β-induced cell cycle arrest in the G0/G1 phase in vulvar squamous cell carcinoma, which enhanced cancer cell apoptosis simultaneously by increasing the expression of apoptotic genes including cleaved caspase-3 [168]. Meanwhile, heteronemin, a natural compound extracted from marine sponges, can reduce the expression of TGF-β1 and intercellular adhesion molecule 1, and inhibits cancer cell adhesion, motility, and proliferation in the bile duct cancer, cholangiocarcinoma [169]. Moreover, a natural bloodroot plant extract sanguinarine can also suppress the proliferation and colony formation of HCC cells via targeting the HIF-1α/TGF-β loop. Primarily, the compound inhibits EMT and the PI3K/AKT pathway and causes, therefore, decreased downstream cancer cell proliferation, migration, and tumour growth [170]. Furthermore, the herbal extract PM014 can also inhibit the generation of pathological lesions in lung diseases, including inflammatory infiltration, fibrosis, and EMT, by repressing TGF-β1 signalling [172]. Furthermore, medical formulas such as “Wenshen zhuanggu” have demonstrated cancer treatment effects via targeting the TGF-β pathway [171]. Some of the above drugs and therapy have been evaluated in clinical trials of cancer treatments. The clinical trials evaluating TGF-β pathway inhibitors are listed in Table 3.

Besides these novel drugs and combinational therapies targeting TGF-β, accurate and effective biomarkers are also required for evaluating and monitoring the effectiveness of these new treatments. Moreover, reliable biomarkers are also necessary for identifying the susceptible and effective population before administering treatment. For example, an increased ratio of CD8+/CD4+ CD3+ T cells and a number of IFN-γ+CD8+ CD3+ T cells can be used to predict enhanced tumour-specific T cell responses in the combination therapy of galunisertib and intestinal microbiota in HCC [173]. Similarly, an increased number of IFN-γ+CD8+ T cells are also an indicator of the successful combination treatment of bintrafusp alfa and radiotherapy [150]. Pu et al. have identified an integrated immune ratio based on the number of infiltrating CD8+ T cells and FOXP3+ Tregs to predict tumour suppression in the combination immunotherapy of CD25, TGF-β, and PD-1 blockade [174]. In melanoma, the treatment mediated by TGFBR1 inhibitor SB505124 in the presence of IL-12 overexpression demonstrates enhanced immune responses, and NK1.1 is a potential biomarker for increased effector cells and enhanced cancer-killing effects [175]. In the combined therapy of galunisertib and paclitaxel, decreased ALDH+ cancer stem-like cell number also reflects treatment efficiency [176].

Radiotherapy is one of the most frequently employed therapeutic approaches for the treatment of cancer, using high doses of ionising radiation to cause cancer cell death and tumour shrinkage. T cells directed to endogenous tumour antigens have been proposed to be crucial mediators of tumour regression [177]. However, ionising radiation also leads to the release of several cytokines, which include TGF-β that has a negative impact on the effectiveness of radiotherapy on tumour regression [178]. Appropriately, a recent study has shown that antibody-mediated TGF-β blockade using the TGFβ-neutralising mAb 1D11 can efficiently enhance radiation-induced cancer treatment through enhancing cytotoxic CD8+ T cell responses in a mouse breast cancer model [177]. Similarly, this beneficial effect of TGF-β blockade can also be observed in cancer radioimmunotherapy. It has been shown that the TGFβ-neutralising mAb 1D11 can markedly enhance the abscopal effects and overall treatment efficacy of combined radio- and immunotherapy in both mouse breast and colorectal cancer models [178]. Again, this synergistic TGF-β blockade and radioimmunotherapy function is mediated through disabling the TGF-β-mediated suppressive function on the anticancer CD8+ T cells [178]. Together, these findings provide strong evidence of the overwhelming positive effects of TGF-β signalling blockade in cancer chemotherapy and radiotherapy.

In summary, TGF-β signalling displays anti-proliferative and tumour suppressive functions during early tumourigenesis, but tumour cells often become refractory to TGF-β-mediated growth inhibition as cancer progresses. Moreover, at late stages of malignancy, TGF-β also targets the vascular, immune, and fibroblastic cellular components of the TME to promote tumour progression and cancer therapy resistance. Conventional chemotherapy and radiotherapy are the most common first-line treatments for the majority of cancers and function primarily by inducing apoptosis, autophagy and cell cycle arrest in tumour cells. However, resistance to these agents often emerges in patients, leading to suboptimal efficacy and disease relapse. As the development of resistance of cancer cells to chemotherapy and radiotherapy is commonly mediated by TGF-β in the TME, chemotherapy and radiotherapy resistance can therefore be reverted by TGF-β inhibition. However, the dual role of TGF-β in cancer development also indicates that long-term treatment strategies with TGF-β inhibitors might promote tumour initiation and also TGF-β-based therapy resistance. Appropriately, current strategies for the use of TGF-β inhibitors as cancer therapeutics involve predominantly short-term and combinatorial therapies. Ultimately, further novel and innovative anticancer immunotherapeutic strategies will stem from a better understanding of the intricate interactions between the malignant transformation, immune system, and the TME, as well as their relationships with TGF-β signalling. In particular, the diversity of TGF-β regulation and functions in different cell types and settings has also rendered anti-TGF-β therapeutic strategies challenging. In consequence, further research into the role and regulation of the TGF-β signalling molecules and target genes in a context-dependent and cell type-specific manner will allow us to dissect and target specific functions of TGF-β to limit cancer initiation, restrict cancer progression and override treatment resistance.

## 5. Conclusions

Extensive studies conducted over the last three decades have demonstrated that TGF-β is an essential immune-suppressive cytokine. Evidently, TGF-β plays diverse roles in the development and maintenance of the immunosuppressive TME. This implies that targeting TGF-β may lead to effective cancer treatment through modulating immunoresponse in the TME. However, further research is required to fully comprehend the TGF-β signalling pathways and their roles in malignant transformation, the immune system, and the TME. For example, unveiling the comprehensive TGF-β modulation network and cofactors involved in cancer immunity will contribute to the development of novel TGF-β-based cancer immunotherapy strategies.

## Figures and Tables

**Table 1:** Mechanistic effectors of the TGF-β pathway in the TME.

Cell Type	Factors	Cancer Type	TGF-β Function and Effect	Species	Reference
T cell	PD-1	Prostate cancer	Limit T cell activation	Human	[75,76]
	IL-1β	Mesothelioma	Limit differentiation and production of Th9 cells	Mouse	[77]
	IL-4				
	IL-22	Colon cancer	Enhance the production of Th17 cells and cancer progression	Human	[78]
	IL-2	Colon cancer	Inhibit the function of Th17 and prevent dendritic cell (DC) antigen presentation	Mouse	[79]
	VEGFA	Melanoma	Reduce regulatory T cells (Tregs) and enhance effector T cell activation in TME	Mouse	[80]
B cell	PD-L1	Colorectal Cancer	Enhance IgA^+^ B cell production and immuno- suppression	Mouse	[81]
	LAP	Breast Cancer	Increase B cell inflation and inhibit T cell, NK cell proliferation	Mouse	[82]
	IL-15				
NK cell	NKG2D	Melanoma	Reform the phenotype of NK cells and reduce their cancer-killing effect.	Human	[83]
	NKp46	Prostate carcinoma		Human	[83]
	DNAM-1				
	NKp30	Non-small cell lung cancer		Human	[83]
	NKp80				
	CD16				
	ILT-2				
	IL-15	Breast cancer, Prostate cancer	Reduce the metabolism and production of NK cells and decrease the amount of NK cell receptors and the cytotoxic effect of NK cells.	Mouse	[84]
	mTOR				
	Smad3	Lewis lung carcinoma, Melanoma	Reduced NK cell population with limited tumour-suppressive activities, and suppress differentiation of NK cells	Mouse	[85]
	E4BP4				
MDSC	CD39	Non-small cell lung cancer	Limit the myeloid-derived suppressor cell (MDSC) immunosuppressive effect and enhance chemo-protective effects to tumour	Human	[86]
	CD73				
	HIF-α				
	IL-7	Lewis lung carcinoma	Suppress B cell responses to tumour though MDSC	Mouse	[87]
	STAT5				
	iNOS	Melanoma	Enhance the release of ROS and NO in MDSCs and suppress the activity and cancer-killing effect of CD8^+^ T and NK cells	Mouse	[88]
	ARG1				
	Fas	HPV-associated head and neck cancer	Enhance antigen-presenting ability	Human	[89]
	
Macrophage	IFN-α	Breast cancer	Inhibit the production of pro-inflammatory cytokines and cause the M2-like differentiation	Mouse	[90]
	IL-10				
	IL-12				
	Snail				
	IL-6	Gastric cancer	Increase M2 differentiation, lead to the proliferation and migration of tumour cells	Human	[91]
	IL-10				
	STAT3				
	SERPINE1	Non-small cell lung cancer	Maintain TGF-β overexpressed in TME and reduce immunosuppression	Human	[92]
	IL-17				
	RTK	Glioblastoma	Increase M2-polarised tumour-associated macrophage (TAM) infiltration and cancer progression	Human	[93]
	PI3K				
DC	IFN-α	Ovarian cancer	Alter plasmacytoid dendritic cells (pDC) functions in TME and increase recruitment, activation of Tregs	Human	[94]
	TNF-α				
	IL-6				
	PD-L1	Lewis lung carcinoma	Induce Treg expansion in TME	Mouse	[95]
	TNFSF18				
	RIG-I	Hepatocellular Carcinoma	Suppresse the production and function of DCs	Human	[96]
Neutrophil	CXCL5	Hepatocellular Carcinoma	Increase neutrophil recruitment and create a pro-tumour TME	Human	[97,98]
	Kras				
	ALK5	Colorectal Cancer	Create a pro-tumour TME and inhibit T cell activation	Human	[99,100]
	MMP9				

**Table 2:** Cancer immunotherapeutic strategies based on the regulation of the TGF-β pathway.

Treatment	Target	Cancer	Effects	Ref.
SRK-181	TGF-β1	Bladder cancer	Increase CD8+ T cells and decrease immunosuppressive myeloid cells	[145]
Anti-PD-1				
Tranilast	TGF-β	Breast cancer	Induce M1 macrophages and improve checkpoint blockade therapy of anti-PD-1/CTLA-4	[146]
Doxil				
E7-TM	SOX2	Lung cancer	Combine with the blockade of checkpoint molecules and induce T cell responses	[147]
Annexin A5 fusion protein	TGF-β3	Lung cancer	Bind to externalised phosphatidylserine of apoptotic cells and restore the sensitivity to chemotherapy	[148]
SX-682	CXCR1/2	Breast cancer Lung cancer	Enhance T cell infiltration and suppress MDSCs	[151]
Bintrafusp alfa	TGFBR2			
Galunisertib	TGFBR1	Glioma	Enhance the cytotoxicity of CD133 and HER2-specific chimeric antigen receptor (CAR) T cells	[155]
		Breast cancer		
SD-208	TGFBR1	Glioma	Induce the infiltration of immune cells, enhance the immunogenicity of tumour cells, and increase the viability of CD4+ and CD8+ ROR1-specific CAR T cells	[156,157]
		Breast cancer		
SB431542	TGFBR1	Fibrosarcoma	Rescue immunosuppressive state induced by Tregs and regulatory B cells (Bregs) and restore T cell cytotoxicity	[158]
NK-92-S3KD	E4BP4	Liver cancer	Promote IFN-γ production and enhance the cancer-killing activity	[159]
		Melanoma		
NK-92-TN	TGF-β	Liver cancer	Promote IFN-γ production, enhance the cancer-killing activity, and inhibit the cell differentiation from naïve CD4+ T cells to Tregs	[160]
FST	Activin-A	Melanoma	Enhance NK cell proliferation and increase granzyme B production	[161]
RGFP966	Smad7	Glioma	Inhibit histone deacetylase and induce the differentiation of tumour stem cells	[162]
Pirfenidone	TGF-β1	Mesothelioma	Reduce the activity of MAPK/AKT pathway and inhibit cancer cell proliferation and migration	[163]
Cabazitaxel	TGF-β	Prostate	Inhibit the colony formation, proliferation, and migration of cancer cells	[164]
	Caspase-3			
	BCL2			
Retinoic acid	TGF-β	Neuroblastoma	Decrease the retinoid-resistant cancer cell viability	[165]
Kartogenin				
Chidamide	TGF-β	Lung cancer	Suppress TGF-β-induced EMT and cancer cell migration	[166]
Vanadium	TGF-β	Breast cancer	Suppress TGF-β-induced EMT and achieve G0/G1 cell cycle arrest	[167]
Carboplatin		Lung cancer		
Astragaloside IV	TGFBR2	Squamous cell carcinoma	Inhibit proliferation, induce cell cycle G0/G1 arrest and apoptosis in cancer cells	[168]
Heteronemin	TGF-β1	Cholangiocarcinoma	Suppress cancer cell proliferation and inhibit TGF-β pathway	[169]
	COX-2			
	ICAM-1			
Sanguinarine	TGF-β	Liver cancer	Block HIF-1α translocation, hypoxia-induced TGF-β secretion, and TGF-β-induced EMT	[170]
	HIF-1α			
Wenshen Zhuanggu formula	TGF-β	Breast cancer	Inhibit the TGF-β pathway via crosstalks with Notch and IL-6 pathways and suppress cancer cell metastasis	[171]

**Table 3:** Clinical trials of the TGF-β pathway inhibitors for cancer treatment.

Drug	Function	Treatment	Trial No.	Year	Location
Belagenpumatucel-L	TGF-β2 inhibitor	NSCLC	NCT01058785	2003	US
Trabedersen	TGF-β2 inhibitor	Pancreatic neoplasm	NCT00844064	2005	Germany
		Colorectal neoplasm			
		Melanoma			
PF-03446962	ALK1 inhibitor	Solid tumour	NCT00557856	2007	US
					Italy
					Korea
Fresolimumab	TGF-β inhibitor	Glioblastoma	NCT01472731	2011	Netherlands
		NSCLC	NCT02581787	2016	US
Vactosertib	TGFBR1 inhibitor	Solid tumour	NCT02160106	2014	US
Galunisertib	ALK5 inhibitor	Solid tumour	NCT02423343	2015	US
		Prostate cancer	NCT02452008	2016	
		Rectal cancer	NCT02688712	2016	
LY3200882	TGFBR1 inhibitor	Solid tumour	NCT02937272	2016	US
		CRC	NCT04031872	2020	Europe
Bintrafusp alfa	TGF-β trap	Breast cancer	NCT03620201	2018	US, Australia
		HPV-associated cancer	NCT04432597	2020	
		HNSCC	NCT04247282	2020	
		ESCC	NCT04481256	2020	
		NSCLC	NCT04297748	2020	
AVID200	TGF-β1/3 inhibitor	Solid tumour	NCT03834662	2019	US
					Canada
GS-1423	TGF-β trap	Solid tumour	NCT03954704	2019	US
SH3051	ALK5 inhibitor	Solid tumour	NCT04423380	2020	China
BCA101	TGF-β trap	Solid tumour	NCT04429542	2020	US
					Canada
SHR1701	TGF-β trap	Solid tumour	NCT04407741	2020	China
		Lymphoma			

## Abbreviations

ALDH	Aldehyde Dehydrogenases
α-SMA	Alpha Smooth Muscle Actin
Bregs	Regulatory B Cells
CAF	Cancer-associated Fibroblast
CAR	Chimeric Antigen Receptor
CDK	Cyclin-dependent Kinase
CEBPA	CCAAT/Enhancer-binding Protein Alpha
CRC	Colorectal Cancer
CREB	cAMP-response Element Binding Protein
CSCs	Cancer Stem Cells
CTLA-4	Cytotoxic T lymphocyte-associated Protein 4
DAPKs	Death-associated Protein Kinases
DCs	Dendritic Cells
DCST1-AS1	DC-STAMP Domain-containing 1-Antisense 1
DNAM-1	DNAX Accessory Molecule-1
ECM	Extracellular Matrix
EGFR	Epidermal Growth Factor Receptor
EMT	Epithelial-to-Mesenchymal Transition
EOMES	Eomesodermin
ESCC	Esophageal Squamous Cell Carcinoma
eT_RM_	Epithelial Resident Memory T cells
FOXO1	Forkhead Box O1
GOLM1	Golgi Membrane Protein 1
HCC	Hepatocellular Carcinoma
HIF-α	Hypoxia Inducible Factor 1 Subunit Alpha
HNSCC	Head and Neck Squamous Cell Carcinoma
IFN-γ	Interferon-γ
IL	Interleukin
ILC	Innate Lymphoid Cell
intILCs	Intermediate Type 1 Innate Lymphoid Cells
iTregs	Induced Regulatory T cells
LAP	Latency-associated Peptide
LLC	Large Latent Complex
MAPK	Mitogen-activated Protein Kinase
MDSCs	Myeloid-derived Suppressor Cells
MKKs	MAP Kinase Kinases
MMP	Matrix Metallopeptidase
MSCs	Mesenchymal Stem Cells
mTOR	Mammalian Target of Rapamycin
NK	Natural Killer
NKG2D	NK Cell Receptor D
NSCLC	Non-Small Cell Lung Cancer
PD-1	Programmed Cell Death 1
PD-L1	Programmed Death Ligand 1
PDA	Pancreatic Ductal Adenocarcinoma
PI3K	Phosphoinositide 3-kinase
PPM1D	Protein Phosphatase 1D
pDC	Plasmacytoid Dendritic Cell
ROCKs	Rho-associated Protein Kinases
SNP	Single-nucleotide Polymorphism
SOX4	SRY-box Transcription Factor 4
TAMs	Tumour-associated Macrophages
TANs	Tumour-associated Neutrophils
TApDCs	Tumour-associated Plasmacytoid Dendritic Cells
TF	Transcription Factor
TGF-β	Transforming Growth Factor-β
TGFBR1	Type I TGF-β Receptor
TGFBR2	Type II TGF-β Receptor
Th	T Helper Cell
TME	Tumour Microenvironment
TNBC	Triple-negative Breast Cancer
TNF-α	Tumour Necrosis Factor Alpha
Tregs	Regulatory T Cells
VEGFA	Vascular Endothelial Growth Factor A
